# Simultaneous multiwavelength study of the reaction of phenolphthalein with sodium hydroxide

**DOI:** 10.1155/S1463924692000294

**Published:** 1992

**Authors:** K. Y. Tam, F. T. Chau

**Affiliations:** Department of Applied Biology and Chemical Technology Hong Kong Polytechnic Hung Hom Hong Kong China

## Abstract

A photodiode array (PDA) spectrophotometer was used to study the fading reaction of phenolpthalein in dilute sodium hydroxide solution. The principal component analysis (PCA) method was employed to identify the number of light absorbing species in the kinetics system. The target factor analysis (TFA) procedure, coupled with the Broyden-Fletcher-Goldfard-Shanno (BFGS) optimization method, was applied to the observed data to deduce the rate constants and the concentration-time profile of the reaction. The internal referencing method was shown to be essential in improving the quality of data obtained by a single beam PDA spectrophotomer.


Journal of Automatic Chemistry, Vol. 14, No. 5 (September–October 1992), pp. 157–162

### Simultaneous multiwavelength study of the reaction of phenolphthalein with sodium hydroxide

**K. Y. Tam and F. T. Chau***
*Department of Applied Biology and Chemical Technology, Hong Kong Polytechnic, Hung Hom, Hong Kong*

*A photodiode array (PDA) spectrophotometer was used to study the fading reaction of phenolpthalein in dilute sodium hydroxide solution. The principal component analysis (PCA) method was employed to identify the number of light absorbing species in the kinetics system. The target factor analysis (TFA) procedure, coupled with the Broyden-Fletcher-Goldfard-Shanno (BFGS) optimization method, was applied to the observed data to deduce the rate constants and the concentration-time profile of the reaction. The internal referencing method was shown to be essential in improving the quality of data obtained by a single beam PDA spectrophotomer.*

### Introduction

The principal component analysis (PCA) method coupled with target factor analysis (TFA) treatment has become a popular method in chemical analyses [1–5]. Based on a proposed reaction model, absorption spectra of the constituting components can be determined [3,5,6]. In this work, the reversible reaction of phenolphthalein with dilute sodium hydroxide solution [7] was studied using a photodiode array (PDA) spectrophotomer. The internal referencing technique [8] was used to pre-possess the spectral data obtained. PCA and TFA (PCA-TFA) methodology was then applied to deduce the concentration-time profile, the absorption spectrum of the reactant, and the rate constants of the reaction. Since the spectral data were acquired at different wavelengths near the absorption maximum with a PDA instrument, the kinetic parameters of the reaction can be derived with higher accuracy than those from spectral information based on only one wavelength by using a scanning spectrophotometer. In this study, it is confirmed that only one visible light absorbing component is present in the phenolphthalein reaction. In addition, the optimized rate constants agrees well with those reported in the literature [7].

### Description of the PDA spectrophotometer

The PDA spectrophotometer developed for this work is shown in figure 1. A Model 6258, 300 Watt Xe lamp, coupled with an Oriel Model 66086 arc lamp source with a power supply, were used to provide UV-Visible light for the absorption study. The light beam generated from the lamp was focused by a fused silica condenser lens (Oriel Model 66013) inside the lamp housing. UV grade fused silica fibre-optic cables, F1 and F2, with diameters of 0·125 inch and numerical aperture of 0·27 (Oriel Model 77564) were used to transmit light to and from the Oriel Model 3089 thermostattable sample compartment respectively. The sample holder was connected to a themostatic circulating bath. One end of F2 was connected to a thermostatic circulating bath. One end of F2 was connected to an Oriel Model 77200, 0·25 m spectrograph with 1200 lines/mm grating of blaze wavelength 500 nm. Absorption spectra were recorded by an Oriel Model 77110 InstaSpec 1B 1024-element PDA detector system [9,10]. Signals from the PDA device were then digitized by a DT-2801-A analogue-to-digital converter card and transferred to an IBM PC/AT. With the present spectrophotometer configuration, the spectral range and resolution of spectra obtained are about 80 nm and 0·2 nm respectively. INSTASPEC (vl.53) software used to acquire spectra and store information on disk [10].

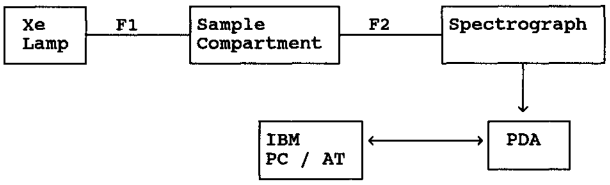

*Figure 1. The PDA spectrometer. F1 and F2 represent the fibre-optic cables and PDA denotes a photodiode array detector system.*

### Description of the method

*Reaction of phenolpthalein with sodium hydroxide*

When phenolpthalein reacts with dilute sodium hydroxide, the process can be described mainly as reversible reaction of the coloured form $R^{2-}$ of phenolphthalein (2,2-bis (p-hydroxyphenyl) phthalide) [11] with the hydroxyl ion.

$$R^{2-} + OH^- \underset{k_2}{\overset{k_1}{\rightleftharpoons}} ROH^{3-} \qquad (1)$$

where $k_1$ and $k_2$ represent the forward and backward rate constants respectively. If an excess amount of hydroxide ion is used, the reaction becomes a pseudo-first-order reversible reaction with the integrated rate law [12] given as follows:

$$C = C_0\ (k_2 + k'_1 \exp\ (-(k'_1 + k_2)t))/(k'_1 + k_2) \qquad (2)$$

* Correspondence to Dr Chau.

0142–0453/92 $3.00 © 1992 Taylor & Francis Ltd.

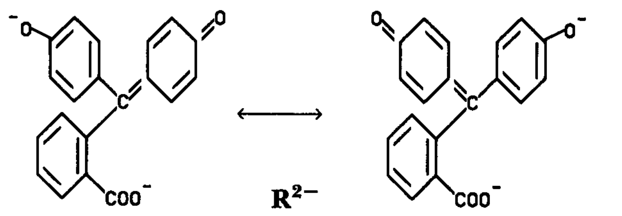

where $C_0$, $t$ and $k'_1$ denotes the initial concentration of phenolphthalein, the time variable and the product $k_1[OH^-]$ respectively.

*Description of the PCA-TFA method*

The PCA and TFA techniques for data treatment have been discussed in detail elsewhere [13,14], and only a brief description of the general aspects is given here. For a kinetics system that is monitored by a PDA spectrophotometer, data obtained are a series of spectra recorded at different time intervals. If *NS* spectra are measured at *NW* wavelengths, the absorbance data collected can be expressed in the form of a matrix as *A* with a dimension of *NS* × *NW*. According to the Beer's law, the absorbance matrix can be written as:

$$A = C\,E \tag{3}$$

where *C* and *E* represent respectively the concentration-time profile of the kinetic system with a dimension of *NS* × *NC* and the absorptivity matrix with a dimension of *NC* × *NW*. *NC* is the number of light absorbing species in the reaction.

The PCA method can be applied to the covariance matrix $A^TA$ with $A^T$ being the transpose of *A*. The eigenvalues $\lambda$ and eigenvectors *O* thus obtained can be divided into two groups. The first group composes of *NC* primary eigenvalues $\lambda_r$ and the corresponding eigenvectors $O_r$ which contain useful information, those in the secondary group are due to noise. Both the *IND* function [13,15] and the eigenvalue ratio (*EVR*) [16] were used in the selection process. The *IND* function is defined as [13,15]:

$$IND = \frac{1}{(NW\text{-}N)^2}\left[\frac{\sum_{j=n+1}^{NW}\lambda(j)}{NS\,(\mathrm{NW\text{-}N})}\right]^{\frac{1}{2}} \tag{4}$$

with $N = 1,2\ldots$; *NW* and $\lambda(j)$ represent the eigenvalue. The function has the minimum value when *N* is equal to *NC*. The *EVR* [16] can be calculated by:

$$EVR(j) = \frac{\lambda(j)}{\lambda(j+1)} \tag{5}$$

with $j = 1,2\ldots$ NW-1

*NC* is equal to $j$-1 when *EVR(j)* is smaller than 7·0.

A mathematically abstract solution of $C_{\mathrm{abs}}$ and $E_{\mathrm{abs}}$ for equation (3) can be obtained from the primary eigenvectors by:

$$C_{\mathrm{abs}} = A\,O_r \text{ and } E_{\mathrm{abs}} = O_r^{\,T} \tag{6}$$

where the superscript *T* represents a transpose operation. A transformation matrix *R* can be generated from a test matrix $C_t$ based on the reaction model proposed in equation (7):

$$1R = \lambda_r^{-1}\,C_{\mathrm{abs}}^{\,T}\,C_t \tag{7}$$

with the superscript −1 denoting an inverse operation. Since reaction (1) was assumed to be a first-order reversible reaction, $C_t$ was evaluated at different time intervals via equation (2) for a given value of $C_o$. With the use of *R*, $C_{\mathrm{abs}}$ and $E_{\mathrm{abs}}$ can be converted to respectively $C_p$ and $E_p$ with physical meanings by the following TFA treatment:

$$C_p = C_{\mathrm{abs}}\,R \tag{8}$$

$$E_p = R^{-1}\,E_{\mathrm{abs}} \tag{9}$$

The *SPOIL* function was suggested [13] to determine whether or not $C_t$ was acceptable. It is defined as the ratio of the real error in the target vector (*RET*) to that in the predicted vector (*REP*) [13,15,17] with:

$$SPOIL = RET/REP \tag{10}$$

where *REP* and *RET* are defined as:

$$REP = \left[\frac{\sum_{j=NC+1}^{NW}\lambda(j)}{NS\,(\mathrm{NW\text{-}NC})}\right]^{\frac{1}{2}}[R\cdot R]^{\frac{1}{2}} \tag{11}$$

and

$$RET = [(AET)^2 - (RET)^2]^{\frac{1}{2}} \tag{12}$$

$R \cdot R$ denotes the dot product of the transformation vector. The apparent error in the test vector (*AET*) can be calculated by:

$$AET = \left[\frac{\sum_{j=1}^{NS}(C_t(j) - C_p(j))^2}{NS}\right]^{\frac{1}{2}} \tag{13}$$

If $C_p$ and $C_t$ give a *SPOIL* function less than 3·0 [15,17,18], $C_t$ is a good description of the concentration profile *C* (equation 3). If not, either alternative values of $k_1$ and $k_2$ should be tested, or the suggested reaction mechanism may not be correct and another needs to be explored. The proposed mechanism can be regarded acceptable if the computational results from TFA satisfy the following acceptance criteria:

(1) The elements inside the $C_p$ and $E_p$ matrices should be positive within experimental error.
(2) The optimized rate constants should be positive and have values which match the reaction time scale.
(3) The calculated absorptivity profiles of reactant and product should be closed to the corresponding experimentally profiles.

$C_t$ can be optimized against $C_p$ by varying the rate constants $k_1$ and $k_2$. Hence the minimization procedure of the *SPOIL* function for $C_p$ and $C_t$ produces an optimization process for the two constants [3,6]. The Broyden–Fletcher–Goldfard–Shanno (BFGS) method, coupled with the Powell's quadratic interpolation linear search technique [19,20], was employed for optimization. A program, FMIND.M, was coded in the PC-MATLAB [21] environment to carry out the computation.

### Experimental

*The reaction of phenolphthalein with sodium hydroxide*

0·1488 g phenolphthalein (Wako) was dissolved in 100 ml 50% aqueous ethanol solution [7]. 1 ml of 3·494 × $10^{-2}$M sodium hydroxide solution was pipetted into a 1 cm glass cell and placed in the thermostattable sample compartment (25·0 ± 0·1 °C) for thermal equilibration. 2 ml of this solution was diluted to 250 ml with water as a working solution and was allowed to equilibrate thermally at 25·0 ± 0·1 °C in a thermostatic bath. 1 ml of phenolphthalein working solution was then pipetted to the glass cell. Mixing of the two reagents was accomplished by using a small magnetic stirrer [22] inside the cell. A magnetic stirrer motor was placed underneath the sample compartment for stirring purpose. In this work, the initial concentration of sodium hydroxide and phenolphthalein were equal to 1·747 × $10^{-2}$ M and 5·952 × $10^{-6}$ per ml respectively. A stop-watch was used to estimate the dead time between mixing of reagents together and the starting time for spectrum acquisition. The dead time was included in the reaction time for subsequent TFA calculations.

The spectral data acquired by the PDA spectrophotometer was calibrated by using emission lines from a sodium lamp. The wavelength accuracy was found to be ±0·6 nm within the spectral range of 536·4 to 608·5 nm. Spectrum acquisition by using the INSTASPEC software was activated as recommended by the manufacturer [10]. The exposure time for each scan was 0·04 s. 201 spectra were recorded every 30 s for 6000s throughout a single experiment.

*Analysis of spectral data*

Each absorption spectrum for the PDA spectrophotometer consists of 1022 data points. It is difficult to use all these data for the PCA-TFA treatment owing to the large computer memory needed and the long computation time required. In addition, absorbance data with low magnitudes are not useful in data analysis. Hence, 10 data points near the absorption maxima of the reaction system with wavelengths of 537·0, 540·4, 543·7, 547·0, 550·3, 553·7, 557·0, 560·3, 563·6 and 566·8 nm were extracted from all spectra measured for PCA-TFA studies. Figure 2 shows a three-dimensional plot of a set of spectral data obtained for reaction (1) at different wavelengths and time intervals with $[R^{2-}]$ = 1·747 × $10^{-2}$ M and $[OH^-]$ = 5·952 × $10^{-6}$ g per ml in 25·0 ± 0·1 °C.

The PDA spectrophotometer used is a single beam device. The fluctuation of light source may give rise to

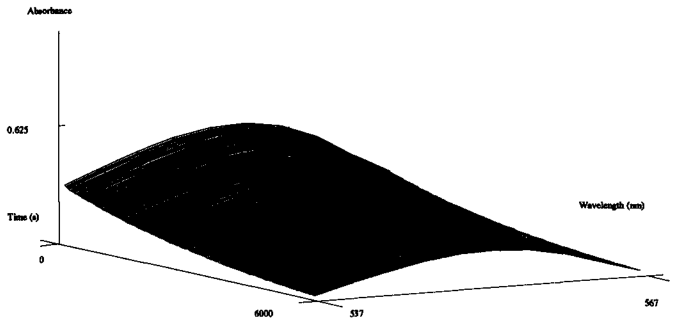

*Figure 2. Three-dimensional plot of a set of spectral data measured by the PDA spectrophotometer for reaction (1) at different wavelengths and time intervals with $[R^{2-}]$ = 1·747 × $10^{-2}$ M and $[OH^-]$ = 5·952 × $10^{-6}$ g per ml in 25·0 ± 0·1 °C.*

erroneous absorbance readings. Since the PDA instrument is capable of acquiring spectral data at different wavelengths almost simultaneously, the internal referencing method [8] can be used to reduce the lamp instability factor on data acquired. In this approach, absorbances obtained for each spectrum in the range of 607·9 to 608·5 nm, where the kinetics system shows no appreciable absorption, were averaged and deducted from those of the 10 wavelengths mentioned above to produce a row of $A$ at a given time interval. Several programs were developed in PC-MATLAB [21] to perform the internal referencing treatment and data extraction (EXTDATA2.M), PCA (AFAE.M), TFA (TTF9E.M). The relative error tolerance [19] adopted in the BFGS optimization process was assigned arbitrarily to $10^{-9}$. A listing of these programs are available from the authors upon request.

### Results and discussion

Table 1 list the results of applying PCA on a typical set of experimental data obtained for the reaction. Both values of the *IND* function and the *EVR* indicate that only one light-absorbing component is present in the kinetic system. Figure 3 gives the differences between $A$ and $A_{abs}$ (= $C_{abs} \times E_{abs}$) at different time intervals for the 10 wavelengths mentioned previously. It can be seen that the residual absorbances distribute randomly for these

*Table 1. Results[a] of PCA study on a set of experimental data for the phenolphthalein fading reaction (1).*

| Factor | IND function | Eigen value ratio |
|---|---|---|
| 1 | *9·3876E-6* | *1·0230E + 6* |
| 2 | 1·0390E-5 | 2·1921 |
| 3 | 1·2855E-5 | 1·1797 |
| 4 | 1·6561E-5 | 1·1138 |
| 5 | 2·2302E-5 | 1·2385 |
| 6 | 3·2584E-5 | 1·5178 |
| 7 | 5·6635E-5 | 1·0352 |
| 8 | 1·2275E-4 | 1·1120 |
| 9 | 4·6348E-4 | 1·2445 |

[a] Values in italics indicate the number of principal components determined by this work.

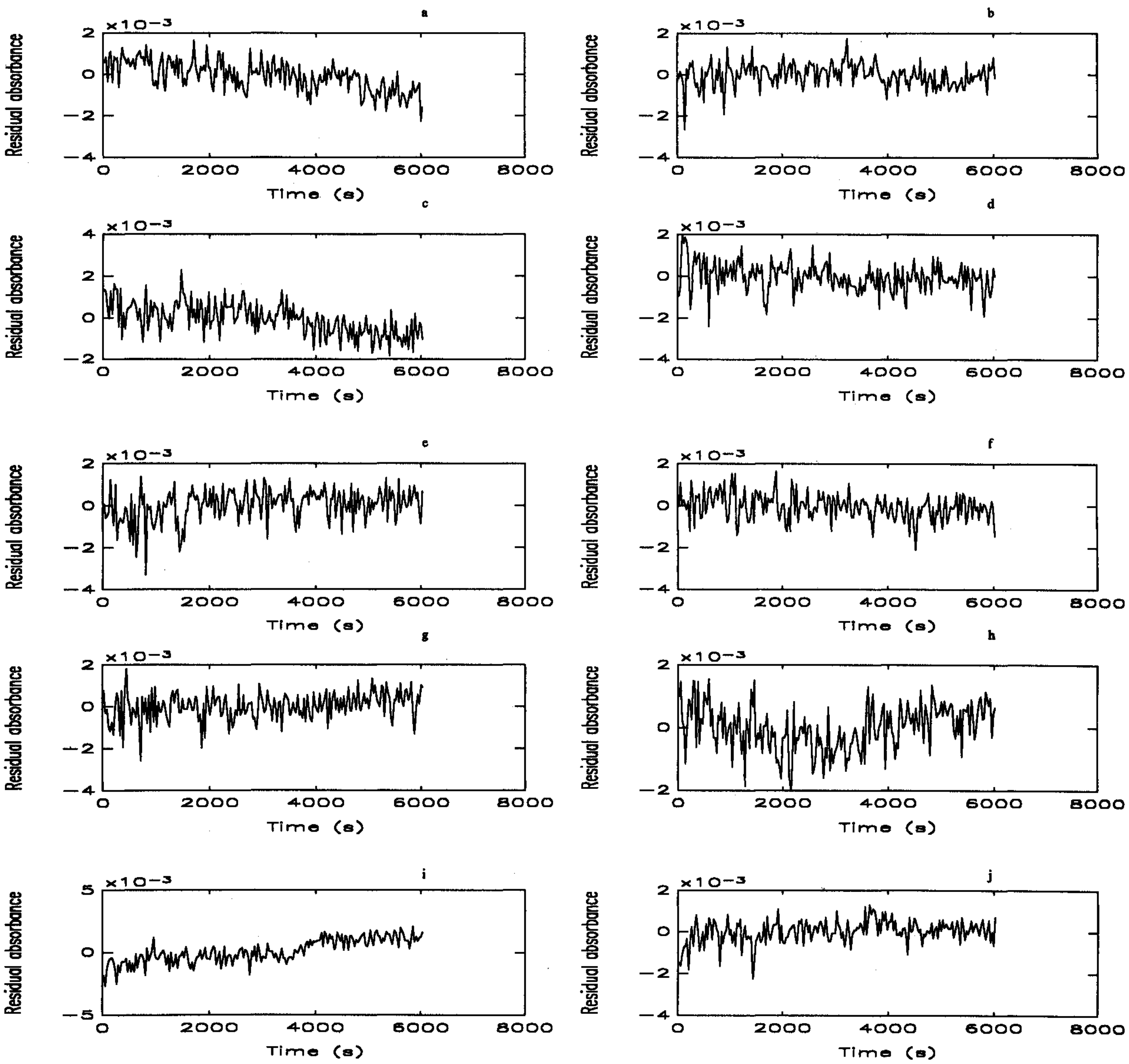

*Figure 3. Plot of the residual absorbance between the experimental (A) and the theoretical ($A_{abs}$) absorbances obtained at 10 analytical wavelengths of (a) 537·0 nm, (b) 540·4 nm, (c) 543·7 nm, (d) 547·0 nm, (e) 550·3 nm, (f) 553·7 nm, (g) 557·0 nm, (h) 560·3 nm, (i) 563·6 nm, and (j) 566·8 nm at different time scales.*

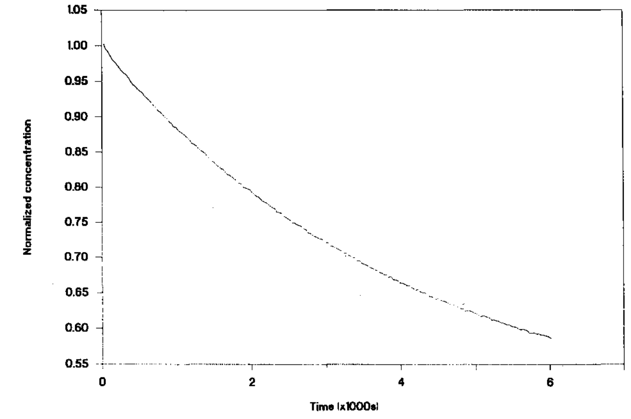

*Figure 4. Normalized concentration-time profiles of $R^{2-}$ in reaction (1) as generated by the PCA-TFA method.*

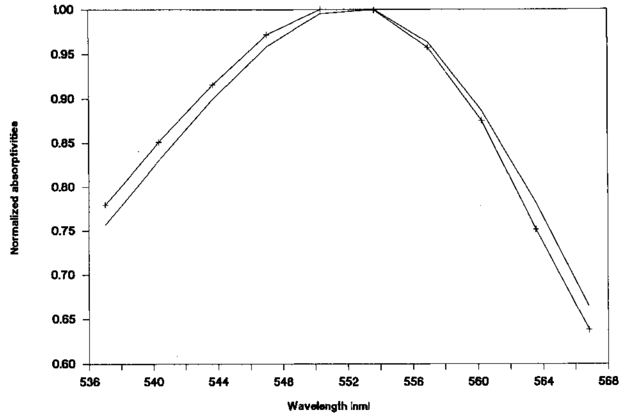

*Figure 5. Normalized absorptivities plots of the phenolphthalein reaction (1) as obtained by a Hitachi U2000 spectrophotometer (—) and derived from the PCA-TFA treatment (-+-+-).*

*Table 2. Rate constants of the pseudo-first-order reversible reaction of phenolphthalein with sodium hydroxide with $[R^{2-}] = 5.952 \times 10^{-6}$ g per ml at 25·0 ± 0·1 °C.*

| | PCA-TFA[a] | | Barners et al.[b] | |
|---|---|---|---|---|
| | With internal referencing | Without internal referencing | [NaOH] = 2·0E-2 M | [NaOH] = 1·6E-2 M |
| $k_1'$[c] ($s^{-1}$) | 7·5114E-3 (±6·1111E-5) | 7·5379E-3 (±1·0642E-4) | 7·9317E-3 | 7·2767E-3 |
| $k_1$ ($M^{-1}s^{-1}$) | 4·2998E-1 (±3·4983E-3) | 4·3150E-1 (±6·0919E-3) | 3·9659E-3 | 4·5479E-1 |
| $k_2$ ($s^{-1}$) | 1·1429E-4 (±1·7269E-6) | 1·1135E-4 (±5·2430E-6) | 1·1170E-4 | 1·0537E-4 |

[a] Rate constants obtained in this work from experimental data with [NaOH] = $1{\cdot}747 \times 10^{-2}$ M. The quantities within parentheses are uncertainties and are equal to three times the standard deviation of the kinetic parameters obtained for three separated measurements.
[b] The reaction was performed in 25 °C and with $[R^{2-}] = 3{\cdot}508 \times 10^{-6}$ g per ml at 25 °C.
[c] $k_1' = k_1$ [OH$^-$] as given in equation (2) – see text.

wavelengths. This further supports that only one light-absorbing component is present in reaction (1).

Figure 4 shows the normalized concentration-time profile ($C_p$) obtained for the phenolphthalein fading reaction using the PCA-TFA method. Figure 5 gives the normalized absorptivity ($E_p$) plots of $R^{2-}$ that were deduced from the TFA treatment and obtained experimentally by a Hitachi U2000 double-beam scanning spectrophotometer. Since the rate of the fading reaction is slow, the scanning spectrophotometer gives the absorption spectrum of the phenolphthalein anion very close to that obtained at the beginning of the reaction. It can be seen in figure 5 that the spectral shapes of the two spectra are similar to each other. This verifies that the PCA-TFA approach is a useful method for extracting absorption spectra of constituent components within a reaction without a prior knowledge of their optical properties. Obviously, for a faster reaction, the present approach with a PDA spectrophotometer is superior to using a scanning spectrophotometer, in terms of obtaining absorption spectra of reaction species. Although the present kinetic system consists of only a single light-absorbing species, the PCA-TFA treatment can be modified easily for cases with many components [6].

Table 2 lists rate constants of the phenolphthalein fading reaction as determined in this work and by Barners *et al.* [7]; is estimated as 1% error. The rate constants extracted by the PCA-TFA method are close to those of Barners *et al.* (within 5%). With internal referencing treatment, the uncertainties of the rate constants are smaller than those without. In all PCA-TFA calculations, all acceptance criteria were satisfied and the *SPOIL* functions had values less than 3·0 for the spectral data with internal referencing pre-processing.

### Conclusion

The pseudo-first-order reversible reaction of phenolphthalein with sodium hydroxide was studied with a PDA spectrophotometer. The internal referencing method was employed first to pre-process absorption spectra obtained. The PCA-TFA method was successfully applied to identify the number of light-absorbing species and to determine the rate constants of the reversible process. Results of this work confirm Barners *et al.* work [7] that only one light-absorbing component is present in the kinetic system. The PCA-TFA method can be extended to multi-component kinetic systems [6] to deduce absorption spectra of intermediates, as well as rate constants of consecutive reactions. In addition, the internal referencing method is found to be essential in improving the quality of spectral data of a single-beam PDA spectrophotometer.

### Acknowledgements

This work was supported by grants from the UPGC of Hong Kong (No. 340/927) and the Research Committee of the Hong Kong Polytechnic (No. 341/510).

### References

1. Halaka, F. G., Babcock, G. T. and Dye J. L., *Biophysics Journal,* **48** (1985), 209.
2. Conchran, R. N. and Horne, F. H., *Analytical Chemistry,* **49** (1977), 846.
3. Billmer, R. I. and Smith, A. L., *Journal of Physical Chemistry,* **95** (1991), 4242.
4. Windig, W., Lippert, J. L., Robbins M. J., Kresinske, K. R. and Twtist, J. P., *Chemometrics Intell. Lab. Syst.,* **9** (1990), 7.
5. Perez-Bendito, D., *Analyst,* **115** (1990), 689.
6. Tam, K. Y. and Chau, F. T., to be submitted for publication.
7. Barners, M. D. and Lamer, V. K., *Journal of the American Chemical Society,* **64** (1942), 2312.
8. Owen, A. J., *Diode-Array Advantage in UV/Visible Spectroscopy* (Hewlett-Packard Co., publication No. 12-5954-8912, Germany, 1988).
9. Oriel Corporation, Light Sources, Monochromators, Detection system (Stratford, 1988).
10. Oriel Corporation, *INSTASPEC 1B Model 77110 Instruction Manual* (Stratford, 1990).

11. Bassett, J., Denney, R. C., Jeffery, G. H. and Mendham, J., *Vogel's Textbook of Quantitative Inorganic Analysis* (Longman, Harlow, 1985).
12. Steinfeld, J. I., Francisco, J. S. and Hase, W. L., *Chemical Kinetics and Dynamics* (Prentice-Hall Inc., Englewood Cliffs, 1989).
13. Malinowski, E. R. and Howery, D. G., *Factor Analysis in Chemistry* (John Wiley & Sons, New York, 1980).
14. Gemperline, P. J., *Journal of Chemometrics,* **3** (1989), 549.
15. Malinowski, E. R., *Analytical Chemistry,* **49** (1977), 612.
16. Woodruff, H. B., Tway, P. C. and Love, L. J. C., *Analytical Chemistry,* **53** (1981), 81.
17. McCue, M. and Malinowski, E. R., *Applied Spectroscopy,* **37** (1983), 463.
18. D'Amboise, M. and Lagarde, B., *Computers and Chemistry,* **13** (1989), 39.
19. Walsh, G. R., *Methods of Optimization* (John Wiley & Sons, New York, 1975).
20. Yuan Y., *IMA Journal of Numerical Analysis,* **11** (1991), 325.
21. The Mathwork, INC., *PC-MATLAB User's Guide* (South Natick 1989).
22. Conrad, R. H., *Analytical Chemistry,* **39** (1967), 1039.

